# Could Total Colectomy with Ileorectal Anastomosis Be an Alternative to Total Proctocolectomy with Ileal Pouch-Anal Anastomosis in Selected Ulcerative Colitis Patients?

**DOI:** 10.1155/2016/5832743

**Published:** 2016-10-23

**Authors:** Francesco Tonelli, Carmela Di Martino, Francesco Giudici

**Affiliations:** Department of Surgery and Translational Medicine, Surgical Unit, University of Florence, Florence, Italy

## Abstract

*Purpose*. To evaluate ileorectal anastomosis (IRA) in selected ulcerative colitis patients.* Methods*. Early and late complications after IRA and IPAA were investigated. Bowel function and quality of life were assessed. Functional and QoL studies were performed as a matched pair analysis, comparing 98 patients who underwent IRA versus 98 patients who underwent IPAA.* Results*. In IRA group, 2 patients (1.6%) developed anastomotic l dysplasia (HGD) developed in 3 patients dysplasia (HGD) developed in 3 patients eakage, 1 patient (0.8%) had intestinal obstruction, and 2 patients (1.6%) had abdominal hematoma. Mean follow-up was 11.5 (range: 2–24.3) years. Failure of IRA occurred in 19 patients (15.1%); in 12 patients (9.5%), failure was related to severe proctitis, in 3 patients (2.4%), it was related to the development of high-grade dysplasia, and in 4 patients (3.2%), it was related to the development of rectal cancer. About functional results, stool consistency [liquid (6.7% of IRA patients versus 29% of IPAA patients; *p* = 0.003)], daily soiling (0% versus 6%; *p* = 0.01), and nocturnal soiling (6% versus 25.5%; *p* = 0.03) were statistically different. Only 1% of IRA patients versus 11% of IPAA patients had episodes of perianal inflammation (*p* = 0.007). CGQoL was 0.72 (±0.14, SD) in IRA patients and 0.75 (±0.11, SD) in IPAA patients (*p* = ns).* Conclusion*. In selected patients, IRA is an appropriate surgical option, with low morbidity, comparable quality of life, and better functional results than IPAA.

## 1. Introduction

Before the introduction of total proctocolectomy with ileal pouch-anal anastomosis (IPAA), total colectomy with ileorectal anastomosis (IRA) was the gold standard procedure to avoid a permanent ileostomy in ulcerative colitis patients (UC patients) [1–5]. The possibility to develop intractable proctitis [6] or dysplasia/carcinoma in the rectal stump [7] was the main criticism of IRA, which was progressively abandoned after IPAA introduction. However, more than thirty-year experience with IPAA highlighted its limits and actually IPAA no longer seems a panacea in UC patients, as several patients have severe postoperative complications and poor functional results [8, 9]. Furthermore, some unsuccessful results after IRA observed in the past could have been due to an improper selection of the patients and a lack of endoscopic and histological surveillance of the rectal stump [7].

Therefore, IRA is to be reconsidered, especially if strict criteria of selection are followed.

We report our experience about UC patients who underwent IRA between 1986 and 2010, analyzing complications, failures, functional results, and quality of life (QoL) after long-term follow-up. Furthermore, we compared the functional results obtained in 98 IRA patients and in a control group of 98 UC patients who underwent IPAA during the same period by the same surgical team.

## 2. Methods

Between January 1986 and December 2010, 137 consecutive UC patients underwent IRA at our centre. Informed consent was obtained from all patients obtaining internal institutional review board approval. Eleven patients (8%) were lost at follow-up. Therefore, the 126 remaining IRA patients scheduled with clinical, instrumental, and laboratory exams performed as outpatient for at least 24 months after IRA were considered.

### 2.1. Indications/Contraindications to IRA

Presence of perianal fistula, damaged anal sphincter, and endoscopically diagnosed severe proctitis were considered contraindications to IRA. Also the occurrence of colorectal severe dysplasia or carcinoma was a contraindication to perform IRA. Immediate intestinal reconstruction was avoided in active severe UC/toxic megacolon. Age, BMI, gender, disease activity in the colon, or perioperative anti-inflammatory medication did not play any role in the decision-making.

From 1996, endoscopy was juxtapose to anorectal manometry, preoperatively performed in all patients to evaluate the rectal status [10]: a maximum tolerated volume (MTV) more than 120 mL air and rectal compliance (RC) more than 1.5 mL air/mmHg were considered as requirements to perform IRA in UC patients; for patients undergoing IRA in two stages, borderline values (within 10% below the cut-off previously indicated) were considered adequate to perform IRA. Therefore, only UC patients with a relatively spared rectum (mild proctitis), appropriate RC and MTV, and normal anal sphincter tone (determined during digital rectal examination or manometry) were considered candidates for an IRA.

### 2.2. Surgical Technique

IRA was performed at the level of the intraperitoneal rectum. The superior hemorrhoidal vessels were preserved. The anastomosis was hand-sewn in a side to end shape. A protective ileostomy was not adopted. When a two-stage IRA was indicated, surgery was performed within six months from the total colectomy to avoid a rectal stump shrunk deteriorating its compliance.

### 2.3. Postoperative Complications and IRA Failure

We evaluated postoperative complications, dividing them as early (within 30 days from surgery) and late (over 30 days after IRA).

IRA failure due to severe proctitis was evaluated differentiating the rate of this complication in patients operated on before and after 1996. The IRA failure was evaluated and compared with the failure rate observed in control group of patients who underwent IPAA.

### 2.4. Therapy and Surveillance of the Rectal Stump

A regular use of rectal topical treatment with suppositories or enemas of mesalazine or corticosteroids was prescribed to all IRA patients in order to prevent or treat a flare-up of the proctitis.

Periodical endoscopies with mucosal rectal biopsies were recommended for the rectum surveillance: once per year in case of UC diagnosed less than 15 years before surgery and every six months when UC was diagnosed more than 15 years before surgery.

### 2.5. Functional Results and QoL

All patients who underwent IRA having more than 24 months of postsurgical follow-up have been invited to answer questionnaires assessing the number of defecation frequencies per 24 h, the stools' consistency (soft or liquid), the day-time and night-time fecal seepage, the ability to distinguish flatus from stools, the episodes of perianal sepsis, the use of perianal protective pads, the need for antidiarrheal medications, antibiotics, systemic 5-ASA, and/or steroids. The assessment of QoL was performed using the Italian version of Cleveland Global QoL (CGQoL) score [11]. We also recorded dietary, social, work, and sexual restrictions. Within the enrolled 126 patients who underwent IRA, 19 patients who developed IRA failure were not included in this investigation: 98 IRA patients agreed to answer the questionnaires and were considered for this analysis. Therefore, functional results and quality of life were also analyzed in a control group of 98 UC patients who underwent IPAA during the same period, operated on by the same surgical team. It was a matched pair analysis matching the patients of IRA and IPAA groups for sex, age, and length of follow-up after surgery, accepting a <5% range tolerance in each value.

### 2.6. Statistical Analysis

The Kaplan-Meier method was used to evaluate IRA and IPAA failure rate. Student's* t*-test and *χ* ^2^ test were used when appropriate and *p* < 0.05 was accepted as a significant value.

## 3. Results

Within the enrolled 126 UC patients who underwent IRA, 96 patients (76.2%) had IRA as one-stage procedure, while 30 patients (23.8%) underwent IRA in two stages. Eighty patients (63.5%) underwent IRA during the period from 1986 to 1995, while 46 patients underwent IRA during the period from 1996 to 2010. Mean follow-up was 11.5 years (range: 2–24.3). The characteristics of the patients are described in Table 1.

During the same period, of a total of 484 UC patients requiring surgery, 309 (68.3%) underwent IPAA, while 38 patients underwent total proctocolectomy with end ileostomy.

### 3.1. Postoperative Complications

No mortality was observed. Early complications affected 5 IRA patients (3.96%): 2 patients (1.6%) developed septic shock due to anastomotic leakage treated with a temporary loop ileostomy; 1 patient (0.8%) showed intestinal obstruction treated conservatively. Two patients (1.6%) had abdominal hematoma, and one of them was surgically treated. Late complications occurred in 4 IRA patients (3.2%): intestinal obstruction requiring adhesiolysis in 1 (0.8%) and anastomotic stricture successfully treated by endoscopic dilatation in 2 (1.6%). Perianal fistulas with intersphincteric abscess treated by drainage and fistulotomy occurred in 1 patient (0.8%).

### 3.2. IRA Failure

**Figure 1:**
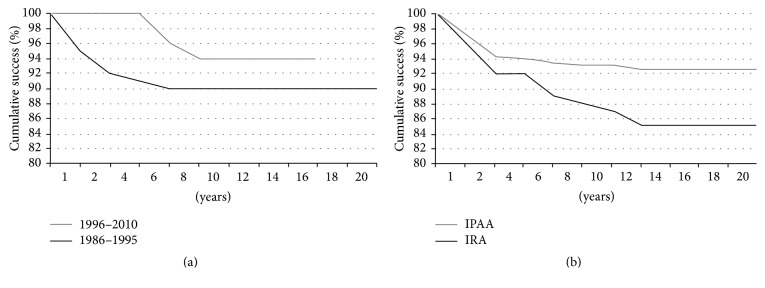
(a) Incidence of IRA failure, due to proctitis, in the two consecutive periods of our experience. A significant difference exists between the two curves (*p* = 0.0047). (b) Cumulative incidence of IRA and IPAA failure during twenty-year period after surgery in our experience.

During follow-up, 19 patients out of 126 (15.1%) had IRA failure. In 12 patients (9.5%), failure was related to severe proctitis with rectal bleeding, diarrhea, and tenesmus not responding to medical therapy. The incidence of the intractable proctitis was significantly higher in our first-period experience (1986–1995), when 9 patients out of 80 (11.2%) required proctectomy for intractable proctitis, while in the following period (1996–2010) proctectomy was needed only in 3 patients out of 46 (6.5%) (*p* = ns). All but one of the 9 patients operated on during 1986–1995 developed proctitis within 5 years from IRA, while no patient operated on between 1996 and 2010 had an early occurrence of severe proctitis (*p* = 0.0047) (Figure 1(a)). All these patients were successfully treated performing IPAA.

Rectal high-grade dysplasia or cancer was the cause of proctectomy in 7 patients. High-grade dysplasia (HGD) developed in 3 patients (2.4%), respectively, at 1, 9, and 13 years from IRA. Four patients (3.2%) developed a rectal cancer (Table 2). The age of patients and the duration of disease at the time of surgery were not significantly related to IRA failure in our patients.

The cumulative failure for patients who underwent IRA is described in Figure 1(b) and it is compared with that observed in UC patients who underwent IPAA; the risk of IRA failure is higher (*p* > 0.05).

### 3.3. Functional Results and QoL

Separately analyzing the 98 IRA patients and 98 IPAA patients who underwent functional evaluation, 36 females (36.7%) were included in IRA group and 38 (38.8%) were included in IPAA group. Mean age at UC diagnosis was 24.3 (range: 16–71) years and 26.3 (range: 13–46) years for IRA and IPAA patients, respectively; mean age at surgery was 35.4 (range: 16–71) years for IRA patients and 36.7 (range: 15–70) years for IPAA patients.

Mean follow-up was 11.5 (range: 2–24.3) years in patients with IRA and 11.3 (range: 2–25.1) years in IPAA patients.

Functional results recorded in IRA and IPAA patients are shown in Table 3.

One IRA patient (1%) and 11 IPAA patients (11.2%) reported skin perianal inflammation episodes (*p* = 0.007).

In order to control diarrhea, 45% of IPAA patients and 46.7% of IRA patients required antidiarrheal drugs (*p* = ns). Eighty-four (85.7%) IRA patients and 65 (66.3%) IPAA patients used systemic steroids (*p* = 0.002). However, in both groups, systemic steroids use was for no more than two cycles per year as mean in order to control flare-up of proctitis or pouchitis and cuffitis, respectively. All the IRA patients (100%) and 77 IPAA patients (78.6%) used cyclically topic steroids (*p* < 0.001). Thirty (30%) IRA patients and no IPAA patients used oral 5-ASA (*p* < 0.001), while all the IRA and IPAA patients used frequently topic 5-ASA. Eighty-eight (89.8%) IRA patients and all IPAA patients needed to cyclically take antibiotics (*p* = 0.01).

Ninety-six (97.9%) IRA patients were satisfied with surgery and they would recommend it to others, while 86 (87.7%) IPAA patients were satisfied with surgery and they would recommend it (*p* = 0.04).

Within IPAA group, the QoL was defined as poor in 12.2%, acceptable in 25.5%, good in 27.5%, and very good in 34.7%, while within IRA group QoL was poor in 3.1% (*p* = 0.001), acceptable in 23.4% (*p* = ns), good in 52% (*p* = 0.01), and very good in 21.4% (*p* = 0.03).

## 4. Discussion

The long-lasting experience with IPAA has shown that there is a consistent risk of postoperative complications and the functional result can be impaired by episodes of pouchitis, cuffitis, or incontinence [8, 9, 12–15]. A recent review of 96 observational studies showed that the IPAA failure rate is 4.7%, analyzing studies published after 2000, and 8.5%, analyzing studies published before 2000 [16].

In the last years, several publications agree that IRA is a safe procedure with a postoperative course usually uneventful and the anastomotic leakage is a rare event [1–5]. The present experience confirms a statistically significant lower risk of both early and late postoperative complications in IRA patients.

About long-term results, many authors had emphasized the disadvantages of IRA due to the preservation of the rectum, with risk to develop intractable proctitis [6, 17] or cancer in the rectal stump [7]. However, these data are not confirmed by more recent experiences [2, 3, 18]. Even if in literature rectal compliance is not a validated parameter for the postoperative development of intractable proctitis, in our opinion it is one of the most important features to indicate IRA. During 1996, we evaluated the preoperative anorectal manometry in relationship with the postoperative functional results [10]. Successively (after 1996), all the UC patients undergoing surgery were selected by preoperative values of MTV and RC: MTV more than 120 mL air and RC more than 1.5 mL air/mmHg were considered as necessary to perform IRA. It is worth noticing that, doing this, IRA failures due to proctitis significantly decreased, being observed only 5 years or more after surgery (Figure 1(a)).

In a recent retrospective study, 22 IRA patients were compared to 66 IPAA patients, showing that the first group had significant lower defecation frequency per day and less night-time seepage but greater urgency and more dietary and work restrictions than the second group; the QoL was similar for both groups [5]. Our study showed that IRA patients have globally a QoL similar to or even better than that of IPAA patients; even if IPAA patients had a better current energy level in CGQoL, social and dietary restrictions were significantly lower in IRA patients, as well as diurnal or nocturnal seepage and perianal inflammation: feces consistence was better in IRA patients. IRA patients needed more frequently local or systemic anti-inflammatory drugs but less frequently antibiotics than IPAA patients. Furthermore, we found a significantly higher rate of patients with poor QoL in the IPAA group compared to the IRA group (12.2% versus 3.1%) but also significantly higher rate of very good QoL in the same IPAA group (34.7% versus 21%).

It is reported that, after IRA, there is a rectal cancer risk that increases with the time elapsed from surgery [17]. It was shown that dysplasia in the rectum progressively increases from 9% at 10 years to 25% at 20 years after IRA [5]. However, the overall incidence of rectal cancer after IRA varies in literature, ranging from 0 to 8% [2, 3, 5, 6, 19]. Lepistö and Järvinen and Leijonmarck et al. reported an incidence of 0%, respectively, in 20 and 51 patients, after a mean follow-up of 18 and 13 years, respectively [2, 6]. Grundfest et al. describe an overall cancer rate of 4.5% at 8-year follow-up [19]. The variability of data regarding the cancer risk after IRA in UC may be due to the presence of colonic carcinoma or dysplasia at the time of surgery: if the IRA is performed in absence of colonic severe dysplasia or cancer, its future occurrence in the rectum is very low (1.3% in a group of 74 IRA patients) [18]. Our data confirmed these evidences.

However, after IRA, a regular endoscopic follow-up with mucosal biopsies is indicated. In our experience, only 3 out of 7 patients who developed rectal HGD or cancer had undergone the scheduled endoscopies, with the occurrence of rectal cancer only in patients who no longer followed scheduled endoscopies and underwent the exam only when symptoms occurred.

## 5. Conclusion

In selected UC patients, IRA can be followed by better functional results than IPAA. If strict criteria of patients' selection are followed, the IRA failure rate is very low and, usually, the failure, for either dysplasia or early cancer or proctitis, does not jeopardize the preservation of the anal sphincter function, thus allowing successful future IPAA.

## Figures and Tables

**Table 1:** Preoperative characteristics and postoperative complications of the 126 UC patients who underwent IRA compared to patients who underwent IPAA during the same period at our centre.

	IRA (126 patients)	IPAA (309 patients)	*p*
Age at surgery (years)			
Mean (range)	35.8 (16–73)	37.5 (14–75)	ns
Sex (M/F)	75/51	161/148	ns
Disease duration (years)			
Mean (range)	6.8 (0–25)	8.4 (0–30)	<0.01
Previous colectomy (%)	23.8	34.6	0.02
MTV ± SD (mL air)	206 ± 77	70 ± 43	<0.01
Rectal compliance ± SD (air/mmHg)	4.8 ± 2.9	3 ± 2.1	0.03
Postoperative complications			
Early: number of patients (%)	5 (4%)	42 (13.6%)	<0.01
Late: number of patients (%)	4 (3.2%)	67 (21.7%)	<0.01
Follow-up (years)			
Mean (range)	11.5 (2–24.3)	10.2 (2–21.4)	ns

IRA: ileorectal anastomosis; IPAA: ileal pouch-anal anastomosis; UC: ulcerative colitis; MTV: maximum tolerated volume; ns: not significant.

**Table 2:** Characteristics of the UC patients with dysplasia or cancer arisen after IRA.

Patient number	Age at IRA	Years from UC diagnosis at IRA	Dysplasia or cancer at colectomy	Regular endoscopic surveillance	Years from IRA to reoperation	Stage	Type of surgery at reoperation	Follow-up from reoperation
1	48	12	—	Yes	13	HGD	IPAA	Alive at 15 yrs
2	35	8	—	Yes	9	HGD	IPAA	Alive at 7 yrs
3	44	21	T2N0	Yes	1	HGD	IPAA	Alive at 5 yrs
4	60	17	HGD	No	12	T3N0	TP + I	Dead after 9 yrs, unrelated causes
5	45	9	—	No	11	T4N1	IPAA	Alive at 5 yrs
6	62	14	HGD	No	13	T3N1	IPAA	Dead after 12 yrs, hepatic Mtx
7	42	10	HGD	No	9	T4N0	TP + I	Dead after 8 yrs, pulmonary Mtx

HGD: high-grade dysplasia; IPAA: ileal pouch-anal anastomosis; TP + I: total proctectomy and terminal ileostomy; Mtx: metastases.

**Table 3:** Functional results, restrictions, and quality of life, according to the Cleveland Global Quality of Life [6], in the two groups of UC patients treated with IRA or IPAA, respectively.

	IRA (98 patients)	IPAA (98 patients)	*p*
Defecation frequency			
Day, mean episodes, number (range)	3.2 (1–7)	4.5 (2–10)	ns
Night, mean episodes, number (range)	0.3 (0–2)	0.87 (0–3)	ns
Consistency of stools (liquid, %)	6.7	29	<0.01
Seepage			
Day, %	0	6	0.01
Night, %	6.1	25.5	0.03
Ability to distinguish flatus/stool %	100	93	ns
Work restriction	6 (6.1%)	7 (7.1%)	ns
Social restriction	27 (27.5%)	40 (40.8%)	0.03
Sexual restriction	1 (1%)	2 (2%)	ns
Dietary restriction	40 (40.8%)	56 (57.1%)	0.03
Current quality of life (mean ± SD)	7.5 ± 1.3	7.3 ± 1.3	ns
Current quality of health (mean ± SD)	7.2 ± 1.5	7.5 ± 1.2	ns
Current energy level (mean ± SD)	7.1 ± 1.5	7.9 ± 1.1	0.045
CGQoL (mean ± SD)	0.72 ± 0.14	0.75 ± 0.11	ns

## Abbreviations

UC: Ulcerative colitis
IRA: Ileorectal anastomosis
IPAA: Ileal pouch-anal anastomosis
QoL: Quality of life
MTV: Maximum tolerated volume
RC: Rectal compliance
HGD: High-grade dysplasia.

## References

[1] Aylett S. (1957). Total colectomy and ileo-rectal anastomosis in diffuse ulcerative colitis. *British Medical Journal*.

[2] Lepistö A., Järvinen H. J. (2005). Fate of the rectum after colectomy with ileorectal anastomosis in ulcerative colitis. *Scandinavian Journal of Surgery*.

[3] Börjesson L., Lundstam U., Öresland T., Brevinge H., Hultén L. (2006). The place for colectomy and ileorectal anastomosis: a valid surgical option for ulcerative colitis?. *Techniques in Coloproctology*.

[4] Felipe Bellolio R., José Miguel Zúñiga A., Pablo Wagner H., George Pinedo M., Ignacio Duarte G., Álvaro Zúñiga D. (2008). Ileorectal anastomosis in the surgical treatment of ulcerative colitis: long-term results. *Revista Medica de Chile*.

[5] Da Luz Moreira A., Kiran R. P., Lavery I. (2010). Clinical outcomes of ileorectal anastomosis for ulcerative colitis. *British Journal of Surgery*.

[6] Leijonmarck C.-E., Löfberg R., Öst Å., Hellers G. (1990). Long-term results of ileorectal anastomosis in ulcerative colitis in Stockholm County. *Diseases of the Colon & Rectum*.

[7] Baker W. N. W., Glass R. E., Ritchie J. K., Aylett S. O. (1978). Cancer of the rectum following colectomy and ileorectal anastomosis for ulcerative colitis. *British Journal of Surgery*.

[8] Fazio V. W., Kiran R. P., Remzi F. H. (2013). Ileal pouch anal anastomosis: analysis of outcome and quality of life in 3707 patients. *Annals of Surgery*.

[9] Hurst R. D., Molinari M., Chung T. P., Rubin M., Michelassi F. (1996). Prospective study of the incidence, timing, and treatment of pouchitis in 104 consecutive patients after restorative proctocolectomy. *Archives of Surgery*.

[10] Tonelli F., Batignani G., Monaci J. (1989). Continenza e modificazioni della pressione del canale e della distensibilità rettale dopo ileorettoanastomosi. *Chirurgia*.

[11] Scarpa M., Ruffolo C., Polese L. (2007). Quality of life after restorative proctocolectomy for ulcerative colitis: different questionnaires lead to different interpretations. *Archives of Surgery*.

[12] Tulchinsky H., Hawley P. R., Nicholls R. J. (2003). Long-term failure after restorative proctocolectomy for ulcerative colitis. *Annals of Surgery*.

[13] Hahnloser D., Pemberton J. H., Wolff B. G., Larson D. R., Crownhart B. S., Dozois R. R. (2007). Results at up to 20 years after ileal pouch-anal anastomosis for chronic ulcerative colitis. *British Journal of Surgery*.

[14] Lepistö A., Luukkonen P., Järvinen H. J. (2002). Cumulative failure rate of ileal pouch-anal anastomosis and quality of life after failure. *Diseases of the Colon and Rectum*.

[15] Romanos J., Samarasekera D. N., Stebbing J. F., Jewell D. P., Kettlewell M. G. W., Mortensen N. J. M. (1997). Outcome of 200 restorative proctocolectomy operations: the John Radcliffe hospital experience. *British Journal of Surgery*.

[16] De Zeeuw S., Ali U. A., Donders R. A. R. T., Hueting W. E., Keus F., Van Laarhoven C. J. H. M. (2012). Update of complications and functional outcome of the ileo-pouch anal anastomosis: overview of evidence and meta-analysis of 96 observational studies. *International Journal of Colorectal Disease*.

[17] Böhm G., O'Dwyer S. T. (2007). The fate of the rectal stump after subtotal colectomy for ulcerative colitis. *International Journal of Colorectal Disease*.

[18] Paoluzi O. A., Di Paolo M. C., Ricci F., Pasquali C., Iacucci M., Paoluzi P. (1994). Ileo-rectal anastomosis in ulcerative colitis: results of a long-term follow-up study. *Italian Journal of Gastroenterology*.

[19] Grundfest S. F., Fazio V., Weiss R. A. (1981). The risk of cancer following colectomy and ileorectal anastomosis for extensive mucosal ulcerative colitis. *Annals of Surgery*.

